# Combined Pharmacotherapy with Alendronate and Desferoxamine Regulate the Bone Resorption and Bone Regeneration for Preventing Glucocorticoids-Induced Osteonecrosis of the Femoral Head

**DOI:** 10.1155/2020/3120458

**Published:** 2020-09-21

**Authors:** Hongfeng Sheng, Yangjun Lao, Shuliang Zhang, Weiguo Ding, Di Lu, Bin Xu

**Affiliations:** Department of Orthopaedics, Tongde Hospital of Zhejiang Province, 234# Gucui Road, Hangzhou 310012, China

## Abstract

**Background:**

Osteonecrosis of the femoral head (ONFH) is a challenge for surgeons and is still without effective treatment method. This study is aimed at evaluating the combined pharmacotherapy with alendronate and desferoxamine for preventing glucocorticoid-induced osteonecrosis of the femoral head (GIOFH) and evaluating the efficacy of the combined medicine in regulating the bone resorption and bone regeneration.

**Materials and Methods:**

Thirty-six rats were randomly assigned to three groups: group A received alendronate and desferoxamine (*n* = 12), group B received alendronate only (*n* = 12), and group C acted as the control group received placebo (*n* = 12). All rats induced the GIOFH using methylprednisolone combined with lipopolysaccharide. Eight weeks later, all rats were killed and their tissues were subjected to radiographic and histological analyses.

**Results:**

According to the results, alendronate administration improved the trabecular thickness and separation in micro-CT analysis but had no significant evidence in increasing the bone area and decreasing the ratio of osteocyte lacunae in histological analysis when compared with the control group. Meanwhile, the alendronate group had more OCs, but less OCN and VEGF levels along with decreased p-AKT, HIF-1*α*, RANKL, and NFATc1 expressions than the control group. For comparison, alendronate combined with DFO further improved the bone volume, trabecular number, trabecular separation, and trabecular thickness with lower ratio of osteocyte lacunae and OC number, higher expression of OCN and VEGF and upregulated signal factors of HIF-1*α* and *β*-catenin, and decreased RANKL and NFATc1.

**Conclusion:**

Combined pharmacotherapy with alendronate and desferoxamine provide significant effects in regulating the bone resorption and bone regeneration for preventing GIOFN.

## 1. Introduction

Osteonecrosis of the femoral head (ONFH) is a common debilitating disease that mostly resulted from alcohol abuse, excessive glucocorticoid (GC) consumption, and hip trauma [1, 2]. ONFH causes hip pain and dysfunction and inevitably progress to femoral head collapse and ultimately requiring total hip arthroplasty (THA) [1–3]. Glucocorticoid-induced osteonecrosis of the femoral head (GIOFH) is the most common type of ONFH, and nearly 50% of patients were steroid induced in China [2]. Up to now, the pathogenesis of GIOFH remains unclear and there is still no effective method to prevent or treat early ONFH [4].

Bone metabolism imbalance induced by GCs is one of the possible pathogenesis for GIOFH [5]. GCs have been shown to increase osteoclast activity and lead to bone loss [5, 6]. When OC leaded bone absorption is greater than osteogenesis, the microstructure and mechanical support component of the femoral head is damaged, resulting in the collapse of the femoral head [5–7]. Bisphosphonate is an effective inhibitor for osteoclast-mediated bone resorption, which can inhibit the nlizophosphorylase in the HMG-COA pathway, thus accelerating the apoptosis of osteoclasts and inhibiting the bone resorption [7, 8]. Therefore, bisphosphonates have been considered a promising medication for early ONFH and preventing femoral head collapse [9–11]. However, studies also indicated that bisphosphonates play a role in reducing bone loss and maintaining the bone volume [12, 13], but it cannot contribute to bone repair [14] and hence has no clinical effects on preventing femoral head collapse or avoiding THA [14, 15]. In fact, bisphosphonates exert significant effects on inhibiting osteoclast activity, but the angiogenesis and osteoblast activity were inhibited, which results in decrease of bone repair [16]. Therefore, the single use of bisphosphonates cannot contribute to the repair of ONFH.

Although core decompression is the most common used method to treat early ONFH in clinic, the pharmacotherapy is still a meaningful and convenient way that attracts much attention [9]. In addition to bisphosphonates, the variety of pharmacotherapy for ONFH has been studied, including statins [9], anticoagulants, and vasodilators [17]. However, none of those medicines could exert significant effects on preventing or repairing ONFH. Deferoxamine (DFO) is a kind of medicine used to treat iron overload-related diseases such as thalassemia. Studies showed that DFO could promote angiogenesis and bone regeneration by upregulating the HIF-1*α*/VEGF pathway and contributing to maintain intracellular homeostasis [18, 19]. Therefore, DFO has been introduced to treat bone defect and osteoporosis [19, 20]. In addition, study reported that DFO promoted angiogenesis and osteogenesis in ONFH and may be a promising choice to treat GIOFH [21]. However, there is still needed study to evaluate the comprehensive curative effect of DFO on ONFN.

In the present study, on the basis of bone homeostasis balance, we aimed to conduct an in vivo study to assess the combined pharmacotherapy with alendronate and desferoxamine for preventing GIOFH and assess whether these two medicines have complementary effects in regulating the bone absorption and bone regeneration and the possible mechanism regarding the RANKL/NFATc1 and HIF-1*α*/*β*-catenin signal pathways.

## 2. Materials and Methods

### 2.1. Animal Treatment and Grouping

Thirty-six 8-week-old male Wistar rats weighing 250-280 g (Animal Center, Zhejiang Academy of Traditional Chinese Medicine) were used in this study. All animals were randomized to three groups with the ratio of 1 : 1 : 1, including group A (alendronate+desferoxamine, *n* = 12), group B (alendronate, *n* = 12), and group C (control, *n* = 12). Group A was administered intragastrically with alendronate (150 *μ*g/kg d) and desferoxamine (250 mg/kg d) (Desferal; Novartis, Switzerland) dissolved in normal saline. Group B was only administered with alendronate (150 *μ*g/kg d), while group C was treated with equivalent normal saline. All animals were fed with 2 weeks before induced GIOFH using the modified method with methylprednisolone (MP) plus lipopolysaccharide (LPS). Briefly, rats received intraperitoneal injection of LPS (0.2 mg/kg, Sigma-Aldrich), then intramuscularly injected with MP 20 mg/kg/d for three continuous days. All animals were fed with study drugs as the three groups show combined with a standard laboratory diet of feed and water until 2 weeks after the final MP injection. All rats were kept to a daily exercise to evaluate the weight-bearing ability of the femoral head. The animal protocol was reviewed and approved by the Animal Care and Committee of our institution.

### 2.2. Micro-CT Scanning

At 8 weeks after final injection of MP, all animals were killed and the bilateral femoral heads were collected and evaluated with a micro-CT scanner (uCT80, vivaCT40; SCANCO Medical AG, Switzerland) at a high resolution and an isotropic voxel size of 19 *μ*m acquired at 0.5° steps over a total rotation of 360° at 80 kV. Three-dimensional (3D) reconstruction and data analysis were accomplished using the built-in software. The following 3D measurement parameters were calculated: bone volume per tissue volume (BV/TV), trabecular number (Tb.N), trabecular separation (Tb.Sp), and trabecular thickness (Tb.Th).

### 2.3. Haematoxylin and Eosin Staining

The femoral head were then decalcified in 20% EDTA solution for about 1.5 months and then dehydrated through a gradient ethanol series, cleared in xylene, and embedded in paraffin. Sections of 4 *μ*m thickness were made and stored in thermostat of 37°C. Specimen sections were stained with haematoxylin and eosin (HE). At last, each piece of the sections was observed by light microscope (BX41, Olympus, Japan) to evaluate the trabecular structure and osteocyte lacunae. Histomorphometric analysis was performed by Image-Pro Plus software (automatic channel measured the total area of red) to measure the bone area (bone area = bone trabecula area/total area of specific area). Five fields were randomly selected in each sample to count the osteocyte lacunae and normal osteocyte (osteocyte lacunae ratio = osteocyte lacunae number/total osteocyte number).

### 2.4. Tartrate-Resistant Acid Phosphatase Staining for Osteoclast

Frozen sections of the decalcified femoral head were used for tartrate-resistant acid phosphatase (TRAP) staining. Tartrate-resistant acid phosphatase (TRAP, Sigma-Aldrich) kit was used to stain the cells following the manufacturer's instructions. The number of positively stained cells was counted from four randomly selected visual fields within the femoral head area [22].

### 2.5. Immunohistochemistry and Immunofluorescence

At the 8th week, immunohistochemistry was used to detect the osteogenic factor osteocalcin (OCN, abcam-93876) and angiogenic factor VEGF (abcam-72807)), while the immunofluorescence was used to detect the molecular of the signal pathways including p-AKT (abcam-222489), HIF-1*α* (abcam-8366), GSK-3*β* (abcam-226169), *β*-catenin (abcam-184919), RANKL (abcam-45039), and p-NFATc1 (MA3-024, Affinity BioReagents). Briefly, the sections were fixed and treated with 50 *μ*g ml−1 4′,6-diamidino-2-phenylindole (DAPI) for nuclear staining. The primary antibodies were diluted to the optimal concentration. The sections were then stained with anti-antibodies and visualized with a secondary antibody conjugated with Cy5 or without Cy5 for immunohistochemistry. Immunohistochemistry images were obtained using a light microscope (BX41, Olympus, Japan). Meanwhile, fluorescence images were acquired using a fluorescence microscope (FluoView 500; Olympus, Tokyo, Japan).

For the semiquantitative analysis of the protein expression, we evaluated the percentage of positive cells (4 scores) and color intensity (3 scores) in immunohistochemistry pictures [23]. Immunofluorescence pictures were analyzed using Image-Pro Plus software to measure protein expression area and cell elongation ratio [24].

### 2.6. Statistical Analysis

Data are presented as mean ± standard deviation (SD). The analysis was performed using SPSS 19.0 software (SPSS Inc., Chicago, IL). One-way analysis of variance (ANOVA) followed by a least significant differences (LSD) *t* test was used to compare the experimental groups. *p* < 0.05 was considered statistically significant.

## 3. Results

### 3.1. Animal General Condition

There were 5 rats that died due to infection or diarrhea before being killed at 8 weeks after final MP administration. Finally, 31 healthy rats were included in our analysis including 10, 10, and 11 in groups A, B, and C, respectively.

### 3.2. Micro-CT Outcomes

**Figure 1:**
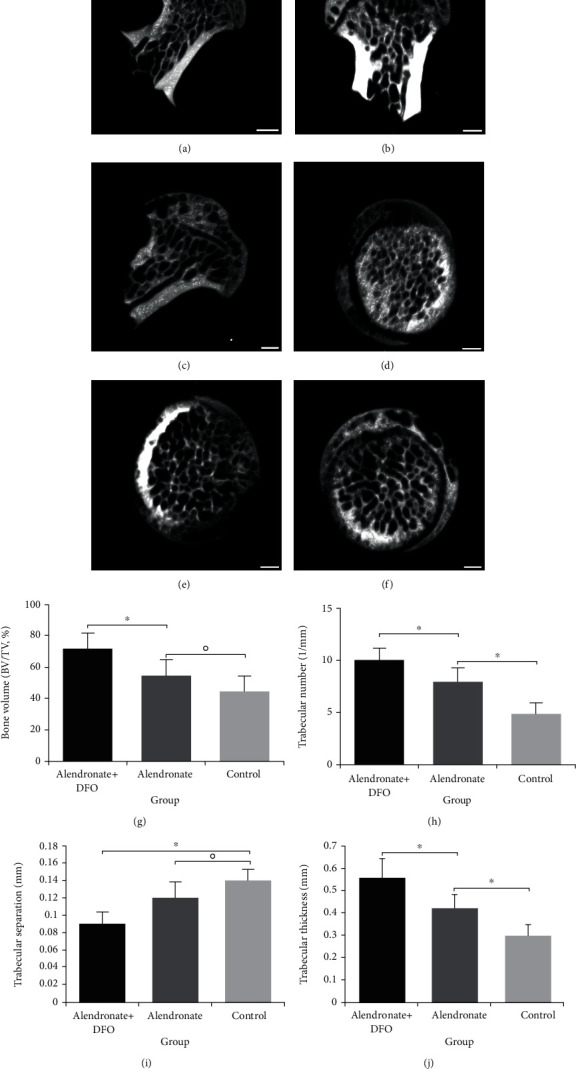
Micro-CT shows the bone trabecular distribution of the femoral head in vertical plane (a–c) and transverse plane (d–f). Scale bar = 1 mm. (a, d) Alendronate and DFO group. (b, e) Alendronate group. (c, f) Control group or placebo group. (g, j) Quantitative outcomes of bone measurement parameters. ^∗^ indicated a significant difference between the two groups (*p* < 0.05). ○ indicated the difference between the two groups without statistical significance (*p* > 0.05).

Figure 1 is the outcomes of micro-CT showing the femoral head in the transverse plane and vertical plane. As is shown, the morphology of the femoral head in the control group was abnormal, and the bone trabecula was sparse and disorganized (Figures 1(c) and 1(f)). While those in the other 2 groups remained relatively regular, but the bone trabecula was also sparse in the weight-bearing area of the femoral head in the alendronate group (Figures 1(b) and 1(e)). For comparison, rats treated with alendronate and DFO obtained compact and regular bone trabecula (Figures 1(a) and 1(d)). When we quantitatively assessed the trabecular bone parameters, we found that the alendronate group provided better performance on trabecular number and trabecular thickness than the control group (*p* < 0.05) (Figures 1(h) and 1(j)), but the bone volume (BV/TV) and trabecular separation were similar between the two groups (*p* > 0.05) (Figures 1(g) and 1(i)). Meanwhile, the rats treated with alendronate and DFO achieved optimal outcomes of the bone volume, trabecular number, trabecular separation, and trabecular thickness among the three groups (*p* < 0.05) (Figures 1(g)–1(j)).

### 3.3. Histological Evaluation

**Figure 2:**
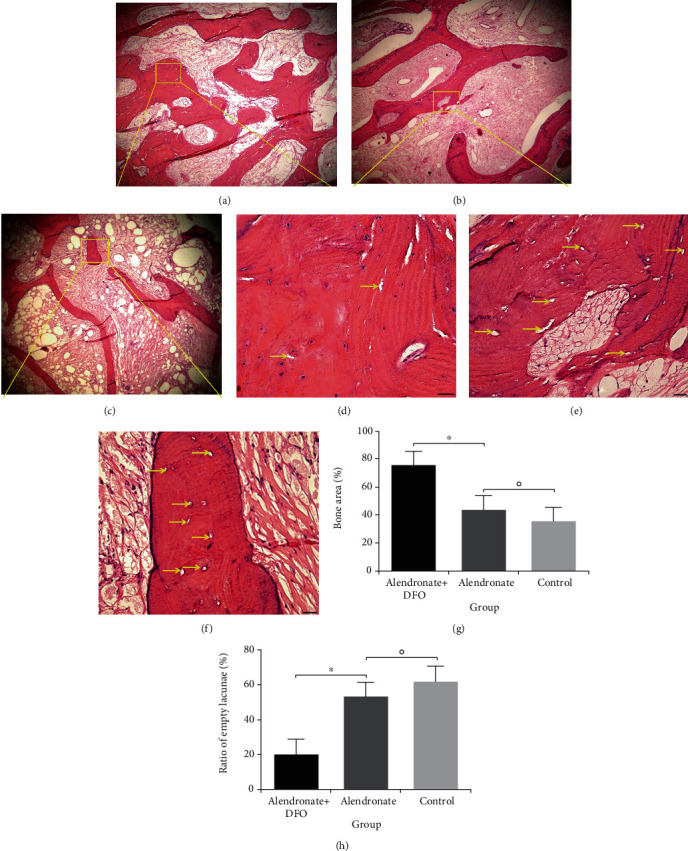
HE staining shows the trabecular structure and osteocyte lacunae of the femoral head ((a–c) scale bar = 200 *μ*m; (d–f) scale bar = 50 *μ*m). Yellow arrow indicated the osteocyte lacunae. (a, d) Alendronate and DFO group. (b, e) Alendronate group. (c, f) Control group or placebo group. (g) Semiquantitative outcomes of bone trabecular area. (h) Quantitative outcomes of the osteocyte lacunae ratio. ^∗^ indicated significant difference between the two groups (*p* < 0.05). ○ indicated the difference between the two groups without statistical significance (*p* > 0.05).

HE staining was used to observe the trabecular morphology and osteocyte lacunae. As is shown in Figure 2, the trabecula is significantly thicker and tighter in group A than the other two groups. In the group A, we only found a small number of osteocyte lacunae. For comparison, group B and group C showed a large area of necrosis on the bone with osteocyte lacunae (Figures 2(a)–2(f)). When we quantitatively assessed the HE images, we found that group A had significant a larger bone area than those in group B and group C (*p* < 0.05), while the latter two groups had similar outcomes (*p* < 0.05) (Figure 2(g)). The ratio of the osteocyte lacunae was 20.4 ± 10.2% in group A, which was lower than group B (52.5 ± 11.0%) and group C (61.2 ± 9.8%) (*p* < 0.05)(Figure 2(h)). Group B and group C had an osteocyte lacunae ratio higher than 50%, which indicated that ONFH had been developed.

### 3.4. Osteoclastogenesis, Osteogenesis, and Angiogenesis Detection

**Figure 3:**
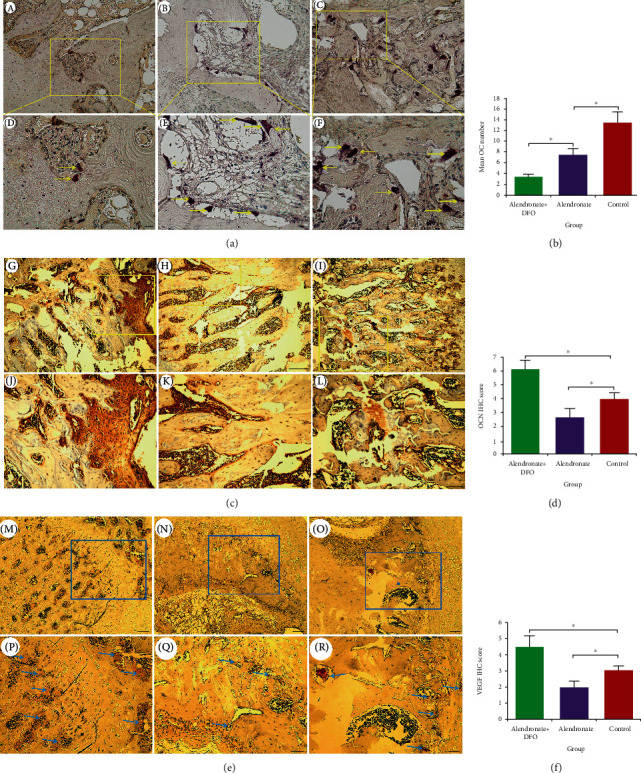
TRAP staining for osteoclasts (a, (A–C) scale bar = 200 *μ*m; (D–F) scale bar = 50 *μ*m). Immunohistochemical outcomes of osteocalcin ((c) (G–I) scale bar = 200 *μ*m; (J–L) scale bar = 50 *μ*m) and VEGF ((e) (M–O) scale bar = 200 *μ*m; (P–R) scale bar = 50 *μ*m) expressions. (b) Quantitative outcomes of OCs number. (d) Semiquantitative outcomes of OCN scores. (f) Semiquantitative outcomes of VEGF scores. ^∗^ indicated significant difference between the two groups (*p* < 0.05).

TRAP staining was used to identify the OC differentiation.  Figure 3(a) showed that the bone trabecula in group A was compact and regular, and only a few TRAP(+) cells can be seen. For comparison, group B had much more TRAP(+) cells with disordered trabecula and physalides, but it was superior to those in group C. When performing the quantitative analysis, group A had least TRAP(+) cells among the three groups and group B had less than group C (*p* < 0.05)(Figure 3(b)).

OCN is a factor indicating the osteogenesis activity. There were a large number of OCN-positive expression cells in group A, which was superior to those in groups B and C (Figure 3(c)). The semiquantitative analysis outcomes also demonstrated that OCN scores were significantly higher in group A than the other two groups (*p* < 0.05) and the scores in group B were also higher than group C (*p* < 0.05) (Figure 3(d)).

VEGF is a key factor for angiogenesis. As is shown in Figure 3(e), groups B and C only showed a little VEGF expression, but group A had a larger area of VEGF-positive expression. At the same time, we also found there were more physalides in groups B and C with more sparse trabecula than group A. The semiquantitative analysis outcomes indicated that VEGF scores in group A was significantly better than those in group C (*p* < 0.05), but group C was higher than group B (*p* < 0.05) (Figure 3(f)).

### 3.5. Immunofluorescence Outcomes of the Signaling Pathway

**Figure 4:**
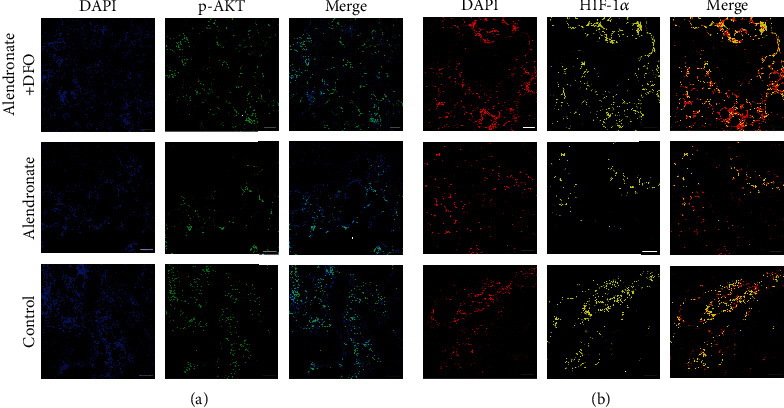
Immunofluorescence images of p-AKT ((a) blue: nucleus; green: p-AKT) and HIF-1*α* ((b) red: nucleus; yellow: HIF-1*α*). Scale bar = 200 *μ*m.

Figure 4 is the IFR images of p-AKT (green) and HIF-1*α* (yellow). As is shown, groups A and C showed a certain amount of positively expressed p-AKT, which was obviously superior to group B. Group B showed a small number of p-AKT-positive expression cells (Figure 4(a)). HIF-1*α* was most expressed in cells of group A and second most expressed in group C, but group B only had little cells that expressed HIF-1*α*.

**Figure 5:**
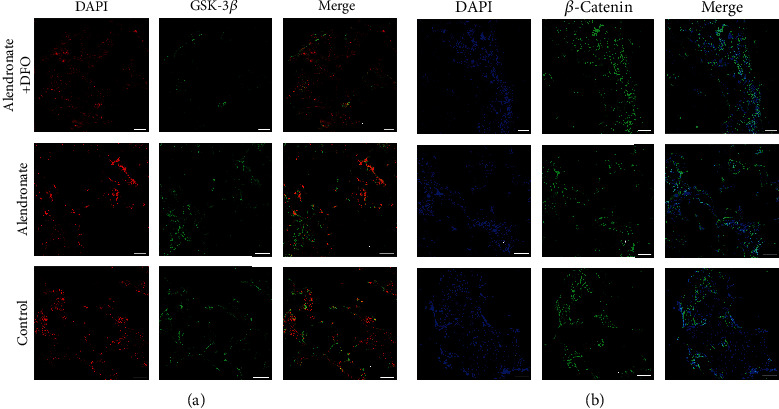
Immunofluorescence images of GSK-3*β* ((a) red: nucleus; green: GSK-3*β*) and *β*-catenin ((b) blue: nucleus; green: *β*-catenin). Scale bar = 200 *μ*m.

Figure 5 shows the expression of GSK-3*β* and *β*-catenin (green) in IFR images. As is shown in Figure 5(a), GSK-3*β* was marginally expressed in group A and seemed to be inferior to groups B and C.  Figure 5(b) demonstrated that group A had positive *β*-catenin expression in most cells, while groups B and C had relative fewer positive expressed cells.

**Figure 6:**
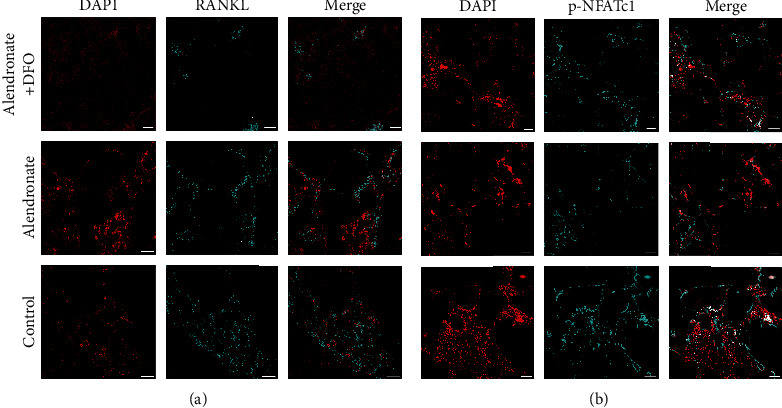
Immunofluorescence images of RANKL ((a) red: nucleus; light blue: RANKL) and NFATc1 ((b) blue: nucleus; light blue: NFATc1). Scale bar = 200 *μ*m.

Figure 6 shows the expression of RANKL and NFATc1 (light blue) in IFR images. Both of these two factors were least expressed in group A, while they were higher expressed in groups B and C. The RANKL in group B seems inferior to those in group C.

**Figure 7:**
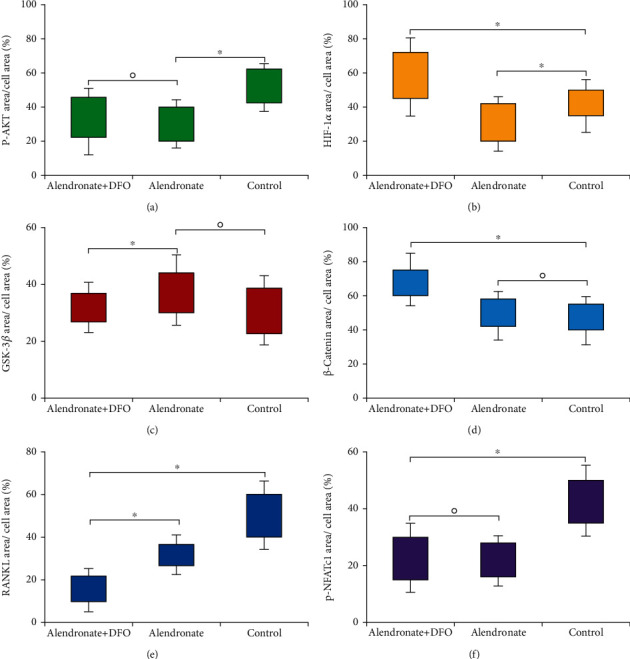
Semiquantitative outcomes of the immunofluorescence images measure the protein expression area. ^∗^ indicated a significant difference between the two groups (*p* < 0.05). ○ indicated the difference between the two groups without statistical significance (*p* > 0.05).

Figure 7 was the semiquantitative analysis of the related protein expression in IFR images. We found that group B had significant lower expression of p-AKT, HIF-1*α*, RANKL, and NFATc1 when compared with group C (*p* < 0.05). Meanwhile, group A showed a higher expression of HIF-1*α* and *β*-catenin (*p* < 0.05) and lower expression of GSK-3*β*, RANKL, and NFATc1 than the other two groups (*p* < 0.05).

## 4. Discussion

OC overactivated is a profound theory in the occurrence and development of GIOFH [25]. In fact, OC is one of the most important factors in bone remodeling, which contributes to absorb the dead bone and promotes bone regeneration [26]. However, with effects of long excessive GCs, OCs are overactivated and the bone loss is increased even exceeding bone formation; therefore, the normal bone structure is destroyed and the femoral head collapsed [27]. Bisphosphonate is effective in inhibiting the activity of OCs and has been used to treat osteoporosis, Paget's disease, and fibrous dysplasia, etc. [13]. However, with the effects of bisphosphonate, the osteogenic factors and angiogenic factors were also decreased, so the bone regeneration and remodeling were inhibited [16, 28]. Therefore, the use of bisphosphonate for treating ONFH is still in controversy. In the present study, we found that the only use of alendronate helped to increase the trabecular thickness and reduce the trabecular separation, but it did not improve the bone area and the ratio of osteocyte lacunae, which indicated that although bisphosphonate played a role in maintaining the bone structure, it lack efficacy in the repair of osteonecrosis. The literature also showed that despite bisphosphonates significantly improving the bone structure in the animal model, no significant efficacy was observed in the treatment of ONFH in the clinical studies [10–15].

We also found that alendronate treatment reduced the OC number, but the OCN and VEGF decreased as well along with lower p-AKT, HIF-1*α*, RANKL, and NFATc1 expressions, which revealed that alendronate downregulated the RANKL/RANK/NFATc1 pathway to inhibit the OC activity, but it had adverse effects on decreasing the activity of the AKT/HIF-1*α* pathway so that the angiogenesis and osteogenesis were affected. In fact, there was a crosstalk between the RANKL/RANK and AKT/HIF-1*α* pathways [29], so alendronate inhibits the ability of RANKL subsequently decreasing the phosphorylation of AKT, resulting in downregulating the osteogenic and angiogenic factor expressions. Consequently, bisphosphonates may provide a performance on inhibiting bone resorption, but the potential adverse effects suppressed the bone regeneration should be taken into consideration.

Long-term use of excessive GCs not only activate OC but also decrease osteogenic differentiation and angiogenesis, all of which lead to the osteonecrosis [1, 4, 21, 23]. As the literature shows, GCs exert effects in downregulating of the wnt/*β*-catenin signal pathway, which inhibits the osteogenic differentiation but increases the adipogenic differentiation, resulting in the occurrence of GIOFH [30]. In addition, HIF-1*α* is a key factor response to ischemia hypoxia and increased in the early GIOFH; hence, the HIF-1*α*/VEGF pathway was upregulated and moreover the downstream factor VEGF was also increased [21, 31]. However, with the continuous effects of GCs, HIF-1*α*/VEGF regulated by the PI3K/AKT pathway was inhibited and the angiogenesis was decreased especially in late ONFH [23, 32]. Fan et al. [33] showed that transplantation of hypoxia preconditioned bone marrow mesenchymal stem cells (BMSCs) could upregulate the HIF-1*α*/VEGF pathway and enhance angiogenesis and osteogenesis in the repair of ONFH. Li et al. [23] reported erythropoietin enhanced bone repair effects via the HIF-1*α*/VEGF signal pathway in GIOFH. Therefore, maintaining the HIF-1*α* expression in the femoral head contributes to the repair of ONFH.

In this study, we used a hypoxia-mimicking medicine (DFO) to reverse the inhabitation effects of alendronate on the AKT/HIF-1*α* pathway and improve the HIF-1*α* activity in the femoral head. The results showed that alendronate combined with DFO reserved the inhibited effect of alendronate on the RANKL/RANK pathway but increased the expression of the HIF-1*α*/VEGF and HIF-1*α*/*β*-catenin pathways, resulting in lower OC differentiation but higher OCN and VEGF expressions. Therefore, alendronate combined with DFO contributed to enhance the bone volume and trabecular number and improved the ratio of osteocyte lacunae in ONFH repair. DFO is clinically used at present, and its safety has been well proved with the specific dosage and administration instructions. According to the literature, 100 mg/kg DFO via intravenous injection has been commonly used [34, 35]. While when administrated via intraperitoneal approach, it can reach 250 mg/kg to 300 mg/kg [21, 36]. In our study, a dosage of 250 mg/kg DFO via intraperitoneal administration is a safe procedure. According to this research, we hope to improve the limitation of bisphosphonate application in the treatment of ONFH in clinic and provide a convenient approach of combined pharmacotherapy for patients at the early stage of ONFH, which may be meaningful to expand the choice of treatment for this disease.

## 5. Conclusion

**Figure 8:**
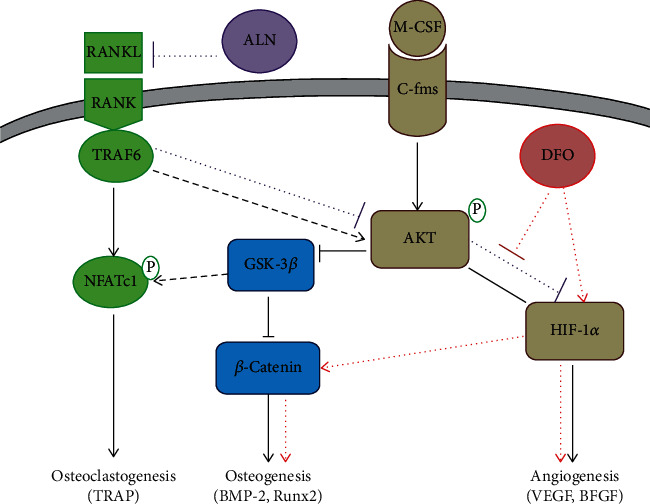
The signal pathway of alendronate and DFO regulating the bone resorption and bone regeneration.

In this work, we demonstrated a significant effect of alendronate on inhibiting the OC regulated bone resorption and maintaining the bone structure in the femoral head, but alendronate has no effects on promoting bone regeneration and even decreased the osteogensis and angiogenesis, which may be associated with decreasing of the RANKL/RANK and HIF-1*α*/*β*-catenin pathways. However, when alendronate combined with DFO was administrated, the bone resorption was also inhibited but the osteogensis and angiogenesis were enhanced along with improved bone volume and empty lacunae, which was controlled by decreased RANKL/RANK while HIF-1*α*/VEGF and HIF-1*α*/*β*-catenin were upregulated (Figure 8). Consequently, combined pharmacotherapy with alendronate and desferoxamine provide significant effects in regulating the bone resorption and bone regeneration for preventing GIOFN.

## Data Availability

The data used to support the findings of this study are available from the corresponding author upon request.
